# Dr. William E. Fitsgibbons

**Published:** 1912-11

**Authors:** 


Dr. William E. Fitsgibbons, Buffalo 1874, died at St. Mary's Hospital, La Salle, Ill., Oct. 3rd, from heart disease, aged 63. He resided at Oglesby, Ill., but had formerly practiced at Wataga and Peru, being health officer at Peru for two years.

